# An exploration of the role of pharmacists within general practice clinics: the protocol for the pharmacists in practice study (PIPS)

**DOI:** 10.1186/1472-6963-12-246

**Published:** 2012-08-09

**Authors:** Edwin Tan, Kay Stewart, Rohan A Elliott, Johnson George

**Affiliations:** 1 Centre for Medicine Use and Safety, Faculty of Pharmacy and Pharmaceutical Sciences, Monash University, 381 Royal Parade, Parkville, VIC, 3052, Australia; 2 Pharmacy Department, Austin Health, Studley Rd, Heidelberg, VIC, 3084, Australia

**Keywords:** Pharmacists, Primary healthcare, General practice, Multidisciplinary, Family practice

## Abstract

**Background:**

Medication-related problems are a serious concern in Australian primary care. Pharmacist interventions have been shown to be effective in identifying and resolving these problems. Collaborative general practitioner-pharmacist services currently available in Australia are limited and underused. Limitations include geographical isolation of pharmacists and lack of communication and access to patient information. Co-location of pharmacists within the general practice clinics is a possible solution. There have been no studies in the Australian setting exploring the role of pharmacists within general practice clinics.

The aim of this study is to develop and test a multifaceted practice pharmacist role in primary care practices to improve the quality use of medicines by patients and clinic staff.

**Methods/design:**

This is a multi-centre, prospective intervention study with a pre-post design and a qualitative component. A practice pharmacist will be located in each of two clinics and provide short and long patient consultations, drug information services and quality assurance activities. Patients receiving long consultation with a pharmacist will be followed up at 3 and 6 months. Based on sample size calculations, at least 50 patients will be recruited for long patient consultations across both sites. Outcome measures include the number, type and severity of medication-related problems identified and resolved; medication adherence; and patient satisfaction. Brief structured interviews will be conducted with patients participating in the study to evaluate their experiences with the service. Staff collaboration and satisfaction with the service will be assessed.

**Discussion:**

This intervention has the potential to optimise medication use in primary care clinics leading to better health outcomes. This study will provide data about the effectiveness of the proposed model for pharmacist involvement in Australian general practice clinics, that will be useful to guide further research and development in this area.

**Trial registration:**

Australian New Zealand Clinical Trials Registry: ACTRN12612000742875

## Background

Medication misadventure remains a serious concern in Australian primary care [1,2]. It is estimated that one in 10 patients who visit their general practitioner (GP) experience a medication-related problem (MRP), of which almost half are considered moderate or severe, with 8% requiring hospitalization [1]. Approximately one in four of these are preventable. These data are consistent with studies from other countries. For example, a recent UK study revealed that one in 20 prescription items in general practice contained an error, affecting 1 in 8 patients [3]. Poor communication has been identified as a major contributing factor towards MRPs, [2] highlighting the need for greater collaboration between GPs, pharmacists and other primary health professionals to ensure optimal patient care.

Collaborative medication reviews, undertaken by pharmacists and GPs, have been successful in identifying and resolving medication-related problems, improving quality of prescribing, and optimizing drug use and costs [4,5]. These collaborative services, however, are currently limited and underused due to factors including geographical isolation, limited access to patient medical records, a lack of time for team activities and a health policy that is not conducive to such collaborative arrangements [6]. Additionally, communication between GPs and pharmacists in the community setting is sporadic and reactive, risking fragmentation of patient care [7].

A possible solution to these problems is the integration of pharmacists into general practices and primary healthcare clinics. In countries such as the United Kingdom, Canada and the United States, practice pharmacists work in close collaboration with GPs (family physicians) to undertake a range of clinical and administrative tasks [8,9]. Some studies suggest the implementation of these roles can result in improved medication use and health outcomes and reduced health service use and cost [10-12]. Co-location has also been shown to enable greater communication and cooperation between health professionals, and strengthen the primary health care team [13].

The aim of this study is to develop and test a multifaceted practice pharmacist role in primary care practices in Australia to improve the quality use of medicines by patients and clinic staff.

## Methods/design

This is a multi-centre, prospective intervention study with a pre-post design and a qualitative component, involving two general practice (primary care) clinics in Melbourne, Australia.

### Recruitment of practices and pharmacists

Primary care clinics will be invited to participate through advertisements and consultation with the Victorian Divisions of General Practice and key informants in Melbourne, Australia. Primary care practices that have the space to accommodate a co-located pharmacist will be targeted. An independent practice pharmacist with significant clinical experience and accreditation to conduct Government funded collaborative Home Medicines Reviews (HMRs) will be identified for each clinic through advertisements and key informants.

### Intervention

The intervention will consist of a multi-faceted, collaborative service targeting patients and practice staff. A practice pharmacist will be co-located in each of the study clinics for at least eight hours per week for six months. The practice pharmacist will undertake the following tasks:

1. Long patient consultations

2. Short patient consultations

3. Drug information and education service for clinic staff; and

4. Quality assurance activities

### Long patient consultation

#### Recruitment of participants

Participants for the long patient consultations will be practice patients who may be at an increased risk of MRPs [14].

#### Inclusion criteria

• Using five or more medicines

• Using one or more medicines that require therapeutic drug monitoring (e.g. warfarin, phenytoin, lithium)

• Using medicines for three or more medical problems

• Have had a recent unplanned hospital admission/emergency department visit

• Having other reason(s) for being at risk of medication misadventure (e.g. adherence issues, language barriers, multiple prescribers)

#### Exclusion criteria

• Have had a HMR in the previous 12 months with no subsequent significant change in clinical status or medication regimen

• Are unable to provide written informed consent

• Are under 18 years of age

• Are unavailable for follow up for six months after recruitment

Patients meeting one or more of the eligibility criteria will be considered for referral to the study by their general practitioner or clinic staff. Those patients who are referred to the study will be provided with an introductory letter and plain language statement in person during their clinic visit. Patients will be asked by the GP/clinic staff if they are willing to be contacted by the research team. If patients agree, the GP/clinic staff will provide the research team with the patient’s contact details.

The research assistant will contact patients who agreed to provide their contact details. Suitable and willing participants will be recruited by obtaining initial verbal consent. Written consent will be obtained at the time of the appointment with the practice pharmacist.

#### Baseline data collection

Baseline data will be collected by the research assistant using a structured questionnaire, either in person (at the clinic or the person’s home) or by telephone, and will include demographic information (age, sex, ethnicity, education, socioeconomic status, living arrangements etc.), health information (general health, health service use, health literacy [15] etc.), and medicines information (medication risk [14] adherence [16,17] etc.). The research assistant will then organize for the participant to meet the practice pharmacist for a long consultation.

#### Long patient consultation process

All participants will receive a 30–60 minute consultation with the pharmacist in a private room at the clinic (or a home visit if they prefer or are housebound) to perform a comprehensive medication review and identify medication-related problems (MRPs). Prior to the consultation, the pharmacist will discuss any health or medication-related issues with the GP or clinic staff, if needed. The pharmacist will also review participants’ general practice medical record (including progress notes, medication lists and pathology results) and dispensing histories if needed. The pharmacist will obtain written informed consent from the participant. In addition to reviewing the participant’s medication regimen, the pharmacist will assess medication adherence and knowledge. The pharmacist will provide counseling and education as needed on medication management and the use of medication devices, and reinforce lifestyle advice related to their health problems and medications. The pharmacist may provide the participant with a complete medication list and refer them to their community pharmacy for adherence aids (e.g. pill boxes, administration aids), if needed. Additionally, referral may be made to the GP or other health professionals as required. After the consultation, the pharmacist will write a short report outlining MRPs and recommendations, and provide this to the participant’s GP either electronically (via secure email or directly into the practice’s electronic medical record) or as a paper-based report, depending on the GP’s preference. The pharmacist may update the medication history within the practice’s medical record as needed. Following this, the pharmacist will discuss any issues with the GP, other staff and community pharmacist, if needed. Case conferencing may be organized where appropriate.

#### Monitoring and follow up

Participants will be followed up by the research assistant at three and six months either in the clinic or by telephone to collect data about implementation of pharmacist recommendations, resolution of MRPs identified by the practice pharmacist, participant’s medication adherence and participant’s general health and wellbeing.

#### Sample size

Based on an expected average number of 2.5 MRPs per participant at baseline, an expected average reduction of 1 MRP (i.e. 40%) per participant with a within-participant standard deviation of 2.1 MRPs (assuming a correlation coefficient of 0.5), [18,19] a power of 80% and a two-tailed alpha of 0.05, the required sample size is 37 participants. Allowing for a dropout/loss to follow-up rate of 25%, at least 50 participants will be recruited. This was calculated using PS Power and Sample Size Calculations (Version 3.0, Dupont & Plummer, 2009).

#### Short patient consultation

Patients with potential medication issues (e.g. nonadherence, newly prescribed medicines) who do not require a comprehensive review of their complete medication regimen may receive a short consultation with the pharmacist. Data will be collected about these consultations, but the patients will not be consented for the study (no personal or identifying data will be collected). Referrals may be made by GPs or clinic staff, or patients may self-refer. The short patient consultation will last 15–30 minutes and provides an opportunity for the pharmacist to provide brief education and counseling on specific needs or answer questions. Short patient consultations will only be undertaken in the clinic. After the consultation, the pharmacist will write brief notes directly into the electronic medical record, and update records as needed. Examples of services to be provided in these consultations include: new medication counseling, adherence assessment, assessment of and education on device technique (e.g. using asthma inhalers), and provision of a medication list. Patient satisfaction will be assessed by an anonymous questionnaire to be given to each patient by the pharmacist along with a reply-paid envelope addressed to the researchers.

#### Drug information and education service

The practice pharmacist will provide a drug information service for practice staff. Queries can be made to the pharmacist in person, by telephone or via email. All queries (and responses) will be documented by the pharmacist. Practice staff will also be invited to attend pharmacist-led group education sessions targeting topics relevant to them (e.g. new therapy or treatment protocols), and a weekly drug information newsletter may also be produced by the pharmacist and provided to staff.

#### Quality assurance activities

The practice pharmacists will undertake a Drug Use and Evaluation (DUE) program in the clinic. The pharmacists will review current prescribing patterns, evaluate these against current best practice guidelines, and implement an intervention to address deficiencies identified using established DUE methodology [20]. A pharmacotherapeutic area of concern or importance will be identified by the pharmacist in conjunction with the GPs and clinic staff. The practice team, with assistance from the research team, will decide upon audit criteria and measurement instruments will be derived from published clinical practice guidelines. The pharmacist will collect and evaluate data relevant to medication use in this area by retrospectively reviewing electronic medical records. Confidentiality of data will be ensured by de-identifying all information collected. Results and feedback will be provided to staff. Multifaceted strategies to improve the quality of prescribing in the selected area will be developed and implemented by the interdisciplinary team, and re-evaluated at the end of the study period. Patients will not be recruited or consented to this part of the study, and no identifying data will be collected.

#### Monitoring and follow up

Actions arising from the DUE program (e.g. development and implementation of clinical guidelines, criteria, treatment protocols, education programs) will be assessed for effectiveness after six months by repeating the prescribing audit to complete the DUE cycle [20].

### Outcomes

#### Outcomes of long patient consultations

##### Medication-related problems

The primary outcome of the long consultations will be the number of MRPs identified by practice pharmacists and the number of identified MRPs that are resolved as a result of the pharmacists’ interventions. The number of MRPs, number of recommendations made by the pharmacists and the number of recommendations accepted/implemented will be recorded and determined by chart audit and/or patient interview, conducted by a research assistant. The types of problems will be categorized by the research team according to the criteria described by Strand et al [21]. The severity of the MRPs and the likely consequences if they had not been addressed will be assessed by an expert panel using a risk classification system [22].

### Other outcomes

#### Satisfaction

Patient satisfaction with the practice pharmacist will be determined by a structured, satisfaction questionnaire adapted from a previously validated patient satisfaction survey by Baker regarding physician consultations [24,25]. The questionnaire will be anonymous and provided at the end of any interaction with the pharmacist (including long patient consultations and short patient consultations). Participants will be requested to complete and return the questionnaire before they leave the practice or at their earliest convenience, using a reply-paid envelope.

#### Quality of prescribing and medication use

The effectiveness of the DUE program will be assessed by re-evaluating medication use in the targeted population at the end of the study and comparing this to baseline data.

#### Drug information queries

The number of drug information queries made and answered, the type of queries made and by whom, will be evaluated at six months.

#### Short patient consultations

General information regarding the nature of the short consultations will be recorded, including the reason for referral, type of service provided and average time spent.

#### Experiences and feedback

The views of a sample of stakeholders on their experiences with this new service will be explored using interviews and/or focus groups at the study’s conclusion. Patients and staff will have the opportunity to share their thoughts on the perceived benefits and challenges of the service and how it could be improved and developed further.

### Data analysis

Data will be entered into the Statistical Package for Social Sciences (SPSS) for Windows Version 19.0 (IBM, New York, USA) and analyzed using standard descriptive methods. Bivariate analysis will be performed between pre- and post-intervention data, using paired t-tests for continuous variables, McNemar chi-square tests for categorical variables, and Wilcoxon matched-pairs signed-ranks tests for ordinal variables. Comparisons between practices may be done using repeated measures analysis of variance (ANOVA). Binary logistic regression may be performed to identify independent predictors of having medication-related problems and effects on adherence and other outcomes.

Qualitative data management will be facilitated using NVivo® (Version 9, Qualitative Solutions & Research International, Melbourne, Vic). Interview transcripts will be read by two independent researchers and coded for emergent themes. Any discrepancies will be discussed and sorted in team meetings in the presence of a third researcher. A framework approach may be utilized whereby a thematic framework, based on *a priori* issues, will be applied to the data [26]. This will allow data to be easily indexed and charted, thus aiding subsequent interpretation.

### Ethics

This study has been approved by the Monash University Human Research Ethics Committee.

## Discussion

To the best of our knowledge, this is the first study in Australia to evaluate an interdisciplinary, multifaceted practice pharmacist role to improve the quality use of medicines by both patients and clinic staff. The comprehensive nature of this intervention aims to optimise medicine use at several levels. Although the study will be conducted with a pre-post design, subjects will serve as their own controls, thus eliminating inter-subject variability and reducing confounding. Additionally, the study will be conducted in more than one primary care clinic allowing for inter-practice comparison and improving external validity.

The study involves mixed methods to capture and measure a variety of data to explain the impact of the intervention. Additionally, a range of process, health, medication management and humanistic outcomes will be collected. By using both quantitative and qualitative methods, a deeper exploration of the intervention can occur. This will enable a more comprehensive exploration of the various intricacies involved in both types of settings, thus allowing identification of the optimal models of pharmacist integration for various primary care clinics.

### Limitations

The before and after design, which lacks a concurrent control group, may compromise the internal validity of the study and limit the conclusions drawn from the results. Non-random sampling means external validity is also compromised. However, this design is the most suitable and practical given the nature of the intervention; a controlled trial with randomization at the level of the patient would be severely limited by contamination, and a cluster randomized controlled trial would require multiple practices, which is not feasible in the context of limited resources.

## Conclusion

The integration and co-location of a practice pharmacist into Australian primary healthcare clinics is uncommon and has not been evaluated. This study will implement and evaluate a new collaborative pharmacist role and assess its effects on optimizing medication outcomes at various levels. The study will provide useful data to guide further research and development in this area.

## Abbreviations

MRP: Medication-related Problem; GP: General Practitioner; HMR: Home Medicines Review; DUE: Drug Use Evaluation; TABS: Tool for Adherence Behaviour Screening; BBQ: Beliefs and Behaviour Questionnaire.

## Competing interests

The authors declare that they have no competing interests.

## Authors’ contributions

ET (PhD candidate) participated in the design of the trial, recruitment and manuscript preparation, and has an ongoing role in carrying out the trial. JG, RAE and KS participated in the design of the trial and study methodology, and review of the manuscript. All authors read and approved the final manuscript.

## Pre-publication history

The pre-publication history for this paper can be accessed here:

http://www.biomedcentral.com/1472-6963/12/246/prepub
